# Prognosis of cancer survivors: estimation based on differential equations

**DOI:** 10.1093/biostatistics/kxab009

**Published:** 2021-09-24

**Authors:** Pål C Ryalen, Bjørn Møller, Christoffer H Laache, Mats J Stensrud, Kjetil Røysland

**Affiliations:** Department of Mathematics, EPFL, Station 8, CH-1015 Lausanne, Switzerland; Department of Registration, Cancer Registry of Norway, Ullernchausseen 64, 0379 Oslo, Norway; Department of Registration, Cancer Registry of Norway, Ullernchausseen 64, 0379 Oslo, Norway, and Department of Biostatistics, University of Oslo, 1122 Blindern, 0317 Oslo, Norway; Department of Mathematics, EPFL, Station 8, CH-1015 Lausanne, Switzerland; Department of Biostatistics, University of Oslo, 1122 Blindern, 0317 Oslo, Norway

**Keywords:** Cancer, Cancer survivors, Conditional survival, Prognosis, Survival analysis

## Abstract

We present a method for estimating several prognosis parameters for cancer survivors. The method utilizes the fact that these parameters solve differential equations driven by cumulative hazards. By expressing the parameters as solutions to differential equations, we develop generic estimators that are easy to implement with standard statistical software. We explicitly describe the estimators for prognosis parameters that are often employed in practice, but also for parameters that, to our knowledge, have not been used to evaluate prognosis. We then apply these parameters to assess the prognosis of five common cancers in Norway.

## 1. Introduction

Many cancer patients would like to learn about their prognosis. Therefore, it is crucial that health care providers give adequate predictions about future outcomes that are relevant and intelligible to the patients. For this purpose, summary estimates on the rate scale, such as hazard ratios, are unlikely to be adequate: patients are unlikely to be interested in the rate of events (e.g., death) in infinitesimal intervals, as provided by estimates on the hazard scale. Furthermore, the estimation of hazard ratios typically relies on parametric assumptions on how the hazard ratio varies over time, such as proportional hazards.

The survival function can easily be estimated without imposing these parametric assumptions but does not necessarily help determine the prognosis of patients that have survived a given time after diagnosis. For example, a prostate cancer patient who has survived 3 years after diagnosis may not be interested in the predicted prognosis from the time of diagnosis: the fact that the patient has survived 3 years may be important for future predictions of life expectancy. Thus, parameters that are explicitly based on the cancer survivors at a given time, such as the conditional 5-year survival function, can be more appropriate. Indeed, several studies have used conditional 5-year survival functions for studying the prognosis of cancer survivors (Janssen-Heijnen *and others,* 2007, 2010; Merrill, 2018; Wancata *and others,* 2016). Although it is straightforward to formulate many other parameters that target cancer survivors’ prognosis, such parameters are not widely used in practice.

In this article, we consider prognosis parameters for cancer survivors that (i) are easy for patients and doctors to interpret and (ii) can be estimated without relying on excessive parametric assumptions. We consider parameters restricted to the surviving population at each time point, and therefore these parameters are tailored to the patients who have survived up to that time point. We derive generic estimators, and explicitly describe these estimators for each parameter under consideration. The parameters are then used to assess the prognosis of common cancers, using national cohorts from the Cancer Registry of Norway.

The remainder of the article is organized as follows. In Section 2, we introduce different prognosis parameters. In Section 3, we outline the estimation procedure for these parameters. In Section 4.1, we describe the cancer data and the results of our analysis. A discussion is given in Section 5.

## 2. Parameters of interest

Our objective is to define and estimate parameters reflecting notions of prognosis intelligible to cancer patients who have survived a given time $t>0$ after being diagnosed. We acquire measures that are tailored for these subjects by conditioning on survival at $t$. In Section 2.1, we will define parameters restricted to the cancer population, targeting both all-cause mortality and cause-specific mortality. In Section 2.2, we will suggest prognosis parameters that contrast summary measures from the cancer cohorts and the general population.

### 2.1. Parameters confined to the cancer population

#### 2.1.1. All-cause mortality parameters

While the survival function provides relevant information about prognosis for subjects at the time of diagnosis, it is less useful for subjects who have survived a given time after diagnosis. However, these cancer survivors may be interested in the conditional $\Delta$-year survival function, defined as

(1) \begin{align*} CS_c(t + \Delta|t) &= \frac{P(T > t + \Delta) }{ P(T > t)}, \label{eq: Delta year conditional survival} \end{align*}

where $T$ is the (possibly censored) time of death in the cancer cohort. It is the probability of surviving until $t + \Delta$, given survival up to $t$. If $\Delta = 5$, and (1) is equal to $0.9$ at $t$, then the average subject alive at $t$ will have a 90% probability of surviving another 5 years.

Some patients prefer thinking about quantities defined on the time scale rather than the probability scale. The restricted mean residual lifetime from $t$ to $t+\Delta$ could be of interest for these patients. It is defined by

(2) \begin{align*} {\it RMRL}_c(t+\Delta|t) =\frac{\int_t^{t + \Delta} P(T > s) {\rm d}s}{P(T > t)}. \label{eq: Delta year conditional restricted mean residual lifetime} \end{align*}

It can be interpreted as the expected remaining lifetime up to $t + \Delta$, given survival up to $t$. A closely related parameter is the conditional restricted mean time lost, $ {\it CRMTL}(t+\Delta | t)$, given by

(3) \begin{align*} {\it CRMTL}(t+\Delta | t) = \Delta -{\it RMRL}_c(t+\Delta|t), \label{eq: conditional restricted mean time lost} \end{align*}

which is the expected number of years lost for the survivors at $t$ in a $\Delta$-year time horizon. If for example, $\Delta = 15$ and (2) is equal to $12$ at $t$, then the cancer subjects alive at $t$ are expected to live $12$ out of the coming $15$ years. Equivalently, the cancer subjects alive at $t$ are expected to lose $3$ years of life in a time horizon of 15 years, which means that (3) is equal to $3$.

#### 2.1.2. Parameters that use the information on causes of death

Patients may be interested in predictions that explicitly target death due to the cancer under study. We can obtain such predictions if the cohort data contain information on the causes of death. Assuming that we can reliably distinguish between causes of death, in particular, death attributed to cancer (e.g., death by cancer or cancer treatment, as stated in a death certificate) and death from causes that are unrelated to cancer, we can define prognosis parameters that distinguish between death due to cancer and death from other causes: if we let $D=1$ denote death from cancer and $D=2$ death from other causes, we can study

(4) \begin{align*} P(T < t + \Delta, D=1 | T \geq t) \label{eq: cancer-specific risk} \end{align*}

as a function of $t$. Moreover, we can contrast the risk of dying due to cancer with risk of dying due to other causes by studying

(5) \begin{align*} &\frac{P(T < t + \Delta, D=1 | T \geq t)}{P(T < t + \Delta, D=2 | T \geq t)} & \text{(relative scale)} \label{eq: cancer-specific RR} \\ \end{align*}

(6) \begin{align*} &P(T < t + \Delta, D=1 | T \geq t) - P(T < t + \Delta, D=2 | T \geq t) & \text{(absolute scale)}. \label{eq: cancer-specific RD} \end{align*}

Another candidate is

(7) \begin{align*} \frac{P( T < t + \Delta, D=1 | T \geq t)}{ P(T < t + \Delta | T \geq t)}. \label{eq: cancer death proportion} \end{align*}

The parameter (4) is the risk of death due to cancer in the interval $[t, t+\Delta]$ for the subjects that have survived up to $t$, and (5) is the relative risk of dying of cancer versus other causes in a period from $t$ to $t+\Delta$ among cancer survivors at time $t$. Similarly, (6) is the difference between the average $\Delta$-year risk of death due to cancer vs death due to other causes among cancer survivors at $t$. Finally, (7) is the proportion of deaths from cancer up to $t+\Delta$, among individuals who have survived until $t$.

### 2.2. Contrasting cancer cohorts with the general population

Cancer patients may be interested in how their predicted outcomes compare to outcomes in the general population. Contrasts of the $\Delta$-year survival function (1) in the cohort $c$ and the general population $g$, that is,

(8) \begin{align*} &\frac{CS_c(t + \Delta|t)}{CS_g(t + \Delta|t)} \label{eq: Delta year conditional survival ratio} &\text{ (relative scale)} \\\end{align*}

(9) \begin{align*} &CS_c(t + \Delta|t) - CS_g(t + \Delta|t) &\text{ (absolute scale)}, \label{eq: Delta year conditional survival difference} \end{align*}

provide such comparisons.

The terms (8) and (9) are the conditional $\Delta$-year survival ratios and differences at $t$. For example, if $\Delta = 5$, and (8) is equal to $0.9$ at $t$, then the conditional 5-year survival of the cohort is 90 % of the conditional 5-year survival of the general population at $t$. Similarly, if $\Delta = 5$, and (9) is equal to $-0.1$ at $t$, then the conditional 5-year survival of the cohort is 10 percentage points worse than the conditional 5-year survival of the general population at $t$.

Similarly, we can compare restricted mean residual lifetime functions between cancer cohorts and the general population, that is,

(10) \begin{align*} &\frac{{\it RMRL}_c(t+\Delta|t)}{{\it RMRL}_g(t+\Delta|t)} &\text{ (relative scale)} \label{eq: Delta year RMRL ratio} \\ \end{align*}

(11) \begin{align*} & {\it RMRL}_c(t+\Delta|t) - {\it RMRL}_g(t+\Delta|t) &\text{ (absolute scale),} \label{eq: Delta year RMRL difference} \end{align*}

to study the prognosis of the cancer survivors at $t$.

If $\Delta = 5$ and (10) is equal to $0.9$ at $t$, then, in the next 5 years, the cancer subjects alive at $t$ are expected to have 90 % of the expected survival of similar subjects in the general population. If $\Delta = 5$ and (11) is equal to $-1$ at $t$, then the average cancer subject alive at $t$, for the given cohort, is expected to lose 1 year of life compared to the general population in the subsequent 5 years.

## 3. Estimation

We estimate the parameters in Section 2 by estimating summary measures of the cancer cohort and the general population separately. We describe estimation procedures for summary measures of the cancer cohorts in Section 3.1 and of the general population in Section 3.2.

### 3.1. Estimating summary measures for the cancer cohorts

A unified framework for modeling survival parameters based on differential equations is described in. A range of summary measures in survival analysis can be formulated as solutions to ordinary differential equation (ODE) systems, that is, they can be written on the form

(12) \begin{align*} X(t) = X(0) + \int_0^t F\big(X(s) \big){\rm d}A(s), \label{eq:ode parameter} \end{align*}

where $X(0)$ is a vector of initial values, $F = (F_1, F_2,\ldots )$ is a matrix-valued function, and $A$ is a $q$-dimensional vector of cumulative hazard coefficients (Ryalen *and others,* 2018; Stensrud *and others,* 2019).

We will use the formulation (12) to derive general estimators and estimation results: by replacing $A$ and $X(0)$ with consistent estimates $\hat A$ and $\hat X(0),$ we obtain a stochastic differential equation (SDE) plugin estimator for $X$;

(13) \begin{align*} \hat X(t) = \hat X(0) + \int_0^t F\big(\hat X(s-) \big) {\rm d}\hat A(s). \label{eq:sde parameter} \end{align*}

Consistency results for (13) when $\hat A$ is the Nelson–Aalen estimator, or more generally Aalen’s additive hazard estimator has previously been developed (Ryalen *and others,* 2018). In particular, as $\hat A$ then is a consistent estimator for $A$ under the independent censoring assumption (Andersen *and others,* 1993), $\hat X$ is also a consistent estimator for $X$ under independent censoring.

A simple example of a parameter that solves (12) is the survival function $S(t) = 1 -\int_0^t S(s){\rm d}A(s)$, that is, with $X(0) = 1$ and $F(x) = -x$ where $A$ is the marginal cumulative hazard for death. By using the Nelson–Aalen estimator to obtain the cumulative hazard estimates $\hat A$, the estimator (13) yields the Kaplan–Meier estimator expressed as a difference equation. Other examples include the cumulative incidence functions, in which case $A$ is the vector of cumulative cause-specific hazards. Several other examples can be found in Ryalen *and others* (2018).

The root $n$ residual limit process solves a linear SDE, and its covariance can be consistently estimated by

(14) \begin{align*} \begin{split} {\hat V}(t) &= {\hat V}(0) + \sum_{j = 1}^q \int_0^t \Big\{ {\hat V}(s-) \nabla F_j( {\hat X}(s-))^\intercal + \nabla F_j( {\hat X}(s-) ) {\hat V}(s-) \Big\} {d} {\hat A}^j(s) \\ & + n \int_0 ^t F( { \hat X}(s-) ){d}{ [ B ]}(s) F( {\hat X}(s-))^\intercal, \end{split} \label{eq:plugin variance} \end{align*}

where $[B](t)$ is a matrix defined by

$$
\begin{align*}
\Big([B](t)\Big)_{i,j}=\begin{cases}
0, \text{ if } {d}A^i(t) = {d}t \text{ or } {d}A^j(t) = {d}t, \\
\sum\limits_{s \leq t} \Delta \hat A^i(s) \Delta \hat A^j(s), \text{ otherwise.}
\end{cases}
\end{align*}$$

It is easy to estimate $\hat X$ and $\hat V$, for example, using an $\texttt{R}$ package that is freely available (Ryalen *and others*, 2018, see $\texttt{github.com/palryalen/transform.hazards}$]).

We build on theory from Ryalen *and others* (2018) to express estimators of the cancer cohort summary parameters in 2. To this end, we consider smooth functions $H$ on the form $H(X(t), X(t+\Delta))$, where $X$ solves (12), and estimators on the form $H(\hat X(t), \hat X(t+\Delta)),$ where $\hat X$ solves (13). In the Supplementary material available at *Biostatistics* online, we show that the covariance of $H(\hat X(t), \hat X(t+\Delta))$ can be estimated by

(15) \begin{align*} \frac{1}{n} \nabla H(\hat X(t), \hat X(t+\Delta)) \begin{pmatrix} \hat V(t) & \hat V(t,t+\Delta)^\intercal \\ \hat V(t,t+\Delta) & \hat V(t+\Delta) \end{pmatrix}\nabla H(\hat X(t),\hat X(t+\Delta))^\intercal, \label{eq: transformed covariance estimator} \end{align*}

where $n$ is the sample size, $\hat V$ is given in (14), and where $\hat V(t,t+\Delta)$ solves

(16) \begin{align*} \hat V(t,t+\Delta) &= \hat V(t) + \sum_{j=1}^q \int_t^{t+\Delta} \nabla F_j(\hat X(s-)) \hat V(t,s-){\rm d}\hat A^j(s). \label{eq: cross covariance estimator} \end{align*}

An argument justifying the consistency of the estimators $H(\hat X(t), \hat X(t+ \Delta))$, (15) and (16) is given in Section A of the Supplementary material available at *Biostatistics* online (http://www.biostatistics.oxfordjournals.org). We may summarize our estimation procedure as follows: for a given prognosis parameter $Q(t+\Delta | t)$ of interest,

a) Find an $X$ that can be written on the form (12), and an accompanying $H(\cdot,\cdot)$, such that $Q(t+\Delta | t) = H(X(t),X(t+\Delta))$.
b) Define the plug-in estimator of $Q$, $\hat Q(t + \Delta | t) = H(\hat X(t), \hat X( t+ \Delta))$, where $\hat X$ is given by (13).
c) Estimate the covariance of $\hat Q$ using (15).

We emphasize that the computations in steps a)–c) can be evaluated generically using a computer. We will nevertheless perform each step for obtaining estimators for the conditional $\Delta$-year survival function in Section 3.1.1. We display the estimators of the remaining parameters from Section 2 in the subsequent sections. Details of the remaining derivations can be found in the Supplementary material available at *Biostatistics* online.

#### 3.1.1. Conditional $\Delta$-year survival

The conditional $\Delta$-year survival function is defined by ${CS}_c(t+\Delta|t) = S(t+\Delta)/ S(t)$ where $S(t) = P(T > t)$ is the (all-cause) survival function in the cancer cohort. As noted in Section 3.1, $S$ solves (12), and by introducing $H(x,y) = y/x$ we get that ${CS}_c(t+\Delta|t) = H(S(t),S(t+\Delta))$. This completes the above step a). Next, calculate $\hat S$, the solution to (13). The estimator defined in step b) is then

$$
\begin{align*}
\hat {CS}_c(t+\Delta|t) = H(\hat S(t), \hat S(t+ \Delta)) = \frac{\hat S(t+\Delta)}{\hat S(t)}.
\end{align*}$$

Our estimation procedure yields a ratio of Kaplan–Meier estimators and is therefore equal to the classical estimator for the conditional survival function. Now, for step c), note first that $F(x) =-x$ and $\nabla F(x) = -1$. The equation (14) is thus,

(17) \begin{align*} \hat V(t) = - 2 \int_0^t \hat V(s-){\rm d}\hat A(s) + n \int_0^t \frac{\hat S(s-)^2}{Y(s)} {\rm d} \hat A(s) \label{eq: survival plugin variance} \end{align*}

as $[B](t) = \sum_{s\leq t} (\Delta \hat A(s))^2 = \int_0^t 1/Y(s){\rm d}\hat A(s)$, where $Y(s)$ is the number at risk at time $s$ and $\hat A$ is the Nelson–Aalen estimator. Next, (16) reduces to a recursive equation which, after simplifying, reads $\hat V(t,t+\Delta) = \hat V(t)\hat{S}(t+\Delta)/\hat{S}(t)$. Thus, by calculating $\nabla_{x,y} H(x,y) |_{x=\hat{S}(t), y=\hat{S}(t+\Delta)}$, and inserting the expressions $\hat V(t)$ and $\hat V(t,t+ \Delta)$, we find that the covariance estimator (15) reads

(18) \begin{align*} \frac{1}{n \hat S(t)^2} \bigg\{ \hat V(t+\Delta) - \hat V(t) \frac{\hat S(t+\Delta)^2}{\hat S(t)^2} \bigg\}. \label{eq: conditional survival variance} \end{align*}

#### 3.1.2. Restricted mean residual lifetime

We define $R(t) = \int_0^t S(s){\rm d}s$. The restricted mean residual lifetime can be written as ${\it RMRL}_c(t + \Delta |t) = (R(t+\Delta) - R(t))/S(t)$. The estimator for ${\rm RMRL}c(t+\Delta | t)$ thus reads

$$
\begin{align*}
\hat {{\it RMRL}}_c(t+\Delta|t) = \frac{\hat R(t+\Delta) - \hat R(t)}{\hat S(t)},
\end{align*}$$

where $\hat R(t)$ is obtained by numerical integration of the Kaplan–Meier estimator (see eq. (2) in the Supplementary material available at *Biostatistics* online). The variance estimator (15) of $\hat {RMRL}_c(t+\Delta|t)$ is

(19) \begin{align*} \begin{split} \frac{1}{n} \Bigg\{ & \begin{pmatrix} \frac{\hat R(t+\Delta)- \hat R(t)}{(\hat S(t))^2} & \frac{1}{\hat S(t)} \end{pmatrix} \hat V(t) \begin{pmatrix} \frac{\hat R(t+\Delta)-\hat R(t)}{(\hat S(t))^2} \\ \frac{1}{\hat S(t)} \end{pmatrix} - \begin{pmatrix} \frac{\hat R(t+\Delta)-\hat R(t)}{(\hat S(t))^2} & \frac{1}{\hat S(t)} \end{pmatrix} \hat V(t,t+\Delta)^\intercal \begin{pmatrix} 0 \\ \frac{1}{\hat S(t)} \end{pmatrix} \\ &- \begin{pmatrix} 0 & \frac{1}{\hat S(t)} \end{pmatrix} \hat V(t,t+\Delta) \begin{pmatrix} \frac{\hat R(t+\Delta)-\hat R(t)}{(\hat S(t))^2} \\ \frac{1}{\hat S(t)} \end{pmatrix} + \begin{pmatrix} 0 & \frac{1}{\hat S(t)} \end{pmatrix} \hat V(t+\Delta) \begin{pmatrix} 0 \\ \frac{1}{\hat S(t)} \end{pmatrix} \Bigg\}, \end{split}\label{eq: RMRL variance} \end{align*}

where the expressions for $\hat V(t)$ and $\hat V(t,t+\Delta)$ are shown in (3) and (4) in the Supplementary material available at *Biostatistics* online.

#### 3.1.3. Parameters using cause of death information

The estimator of (4) takes the form

$$
\begin{align*}
\frac{\hat C^c(t+\Delta) - \hat C^c(t)}{\hat S(t)},
\end{align*}$$

where $\hat C^c(t) = \int_0^t \hat S(s-) {\rm d}\hat A^c(s) $, and $\hat A^c$ is the Nelson–Aalen estimator for the cause-specific hazard for death due to cancer. The estimator for (5) reads

$$
\begin{align*}
\frac{\hat C^c(t+\Delta) - \hat C^c(t)}{\hat C^o(t+\Delta) - \hat C^o(t)},
\end{align*}$$

where $\hat C^o(t) = \int_0^t \hat S(s-){\rm d}\hat A^o(s) $, with $\hat A^o$ being the Nelson–Aalen estimator for the cause-specific hazard for death due to other causes. The estimator for (6) is

$$
\begin{align*}
\frac{\hat C^c(t+\Delta) - \hat C^c(t)}{\hat S(t)} - \frac{\hat C^o(t+\Delta) - \hat C^o(t)}{\hat S(t)},
\end{align*}$$

and the estimator of (7) is

$$
\begin{align*}
\frac{\hat C^c(t+\Delta) - \hat C^c(t)}{\hat S(t)- \hat S(t+\Delta)}.\end{align*}$$

The variance estimator for these estimators takes the general form

$$
\begin{align*}
\frac{1}{n} \Big( & a(t)^\intercal
\hat V(t)
a(t) + a(t)^\intercal
\hat V(t,t+\Delta)^\intercal b(t)
+ b(t)^\intercal \hat V(t,t+\Delta)
a(t) + b(t)^\intercal \hat V(t+\Delta)
b(t) \Big),
\end{align*}$$

where, for (4), (5), (6), and (7) respectively, $a(t)$ and $b(t)$ are

$$
\begin{align*}
a(t) &= \frac{1}{\hat S(t)} \begin{pmatrix} - \frac{\hat C^c(t+\Delta)- \hat C^c(t)}{\hat S(t)} & -1 & 0 \end{pmatrix}^\intercal & b(t) &= \frac{1}{\hat S(t)} \begin{pmatrix} 0 & 1 & 0 \end{pmatrix}^\intercal \\
a(t) &= J_1(t) \begin{pmatrix} 0 & -1 & \frac{\hat C^c(t+\Delta)- \hat C^c(t)}{\hat C^o(t+\Delta)- \hat C^o(t)} \end{pmatrix}^\intercal & b(t) &= J_1(t) \begin{pmatrix} 0 & 1 & -\frac{\hat C^c(t+\Delta)- \hat C^c(t)}
{\hat C^o(t+\Delta)- \hat C^o(t)} \end{pmatrix}^\intercal \\
a(t) &= \frac{1}{\hat S(t)} \begin{pmatrix} - \frac{\hat C^c(t+\Delta) - \hat C^o(t+\Delta) - \hat C^c(t)
+ \hat C^o(t)}{\hat S(t)} & -1 & 1 \end{pmatrix}^\intercal & b(t) &= \frac{1}{\hat S(t)} \begin{pmatrix} 0 & 1 & -1 \end{pmatrix}^\intercal
\\
a(t) &= J_2(t) \begin{pmatrix} -\frac{\hat C^c(t+\Delta) - \hat C^c(t)}{\hat S(t)-\hat S(t+\Delta)} & -1 & 0 \end{pmatrix}^\intercal & b(t) &= J_2(t) \begin{pmatrix} \frac{\hat C^c(t+\Delta) - \hat C^c(t)}{\hat S(t)-\hat S(t+\Delta)} & 1 & 0 \end{pmatrix}^\intercal,
\end{align*}$$

and where the expressions for $\hat V(t)$ and $\hat V(t,t+\Delta)$ can be found in (5) and (6) in the Supplementary material available at *Biostatistics* online. Here, we have defined $J_1(t) = 1/(\hat C^o(t+\Delta)- \hat C^o(t))$ and $J_2(t) = 1/(\hat S(t)-\hat S(t+\Delta))$.

### 3.2. Obtaining summary measures for the general population

From Statistics Norway, we have access to yearly all-cause mortality rates, or discrete-time hazards, stratified by calendar year, age, and sex for the general Norwegian population. Heuristically, by matching each subject in the cancer cohort with a (fictitious) subject in the general population, we identify a subgroup of the general population with a similar distribution of demographic variables compared to the cancer cohort. We then obtain the marginal hazard of this subgroup using the Ederer I estimator (Ederer *and others,* 1961), implemented in the $\texttt{rs.surv}$ function in the $\texttt{R}$ package $\texttt{relsurv}$ (Pohar Perme and Pavlic, 2018). Using the population hazard, we calculate the population parameters $CS_g(t+\Delta|t)$ and $RMRL_g(t+\Delta|t)$, applying numerical integration when necessary.

## 4. Analysis of five common cancers

### 4.1. Data

We used data from The Cancer Registry of Norway to consider the five most frequently registered cancers in Norway in 2018: prostate, breast, lung, colon, and melanoma of the skin (Larsen *and others,* 2019). We had access to data on sex, age, date of diagnosis, date of death, and the cause of death. We restricted the analysis to the subjects who were diagnosed in 2001 or later, and who were younger than 60 years at the time of diagnosis. The subjects were administratively censored on December 31, 2018.

### 4.2. Results

**Fig. 1.**
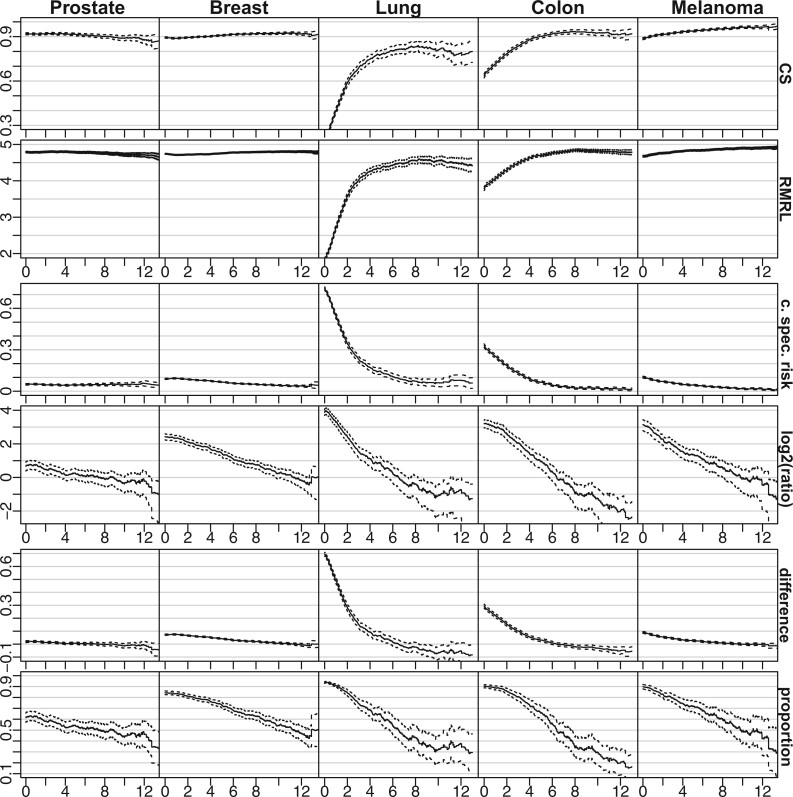
Estimates of the parameters (1), (2), and (4) –(7) (ordered row-wise) with $\Delta = 5$ using the estimators described in Section 3. 95% confidence intervals, obtained using our variance estimator, are indicated with dashed lines. In the uppermost row, we also plotted 95% confidence intervals derived from the Greenwood estimator (dotted lines). Our variance estimates are in close agreement with the classical variance estimates, making the dotted and dashed lines indistinguishable at a glance. This coincides with more extensive comparisons already performed (Stensrud *and others,* 2019). The five cancers are ordered column-wise, and the ticks at the $x$-axes are vertically aligned.

We plot parameters from Section 2.1 in Figure 1 with $\Delta = 5$. For prostate and breast cancer, $CS_c(t+\Delta|t)$ and ${\rm RMRL}_c(t+\Delta | t)$ are roughly constant over time, having values around 0.9 and 4.7, respectively. The lung and colon cancer survivors display a more pronounced improvement; the lung cancer patients at the time of diagnosis are expected to live two out of the coming 5 years, but we expect that the survivors at $t=6$ years to live 4.5 out of the next 5 years. The melanoma patients have a slight improvement from diagnosis up to 12 years, with a 5-year survival of 0.9 at $t=0$ and a conditional 5-year survival over $0.95$ at $t = 12$.

The risk of death due to cancer is relatively constant or moderately declining as time progresses. The proportions of death attributable to cancer and the risk ratios of dying due to cancer versus other deaths are declining more steadily. For instance, the prostate cancer survivors have about a 6% risk of dying due to cancer in a 5-year horizon throughout the study period (from Figure: 95% confidence interval at $t=12$ is [0.04–0.09]). Still, the 5-year risk ratio (5) declines from almost two at $t=0$ to about one at $t = 5$. Thus, prostate cancer patients at diagnosis are about twice as likely to die due to cancer within 5 years, but the survivors at $t=5$ are equally likely to die of other causes in a 5-year horizon. For the other cancers, the conditional 5-year risk of dying due to cancer decreases more steadily over time. Also, the 5-year risk of dying due to other causes than cancer varies between the cancer types. For instance, melanoma and prostate cancer survivors have about a 6% 5-year risk of dying due to cancer after 4 years (from Figure: both have 95% confidence intervals [0.05–0.07] at $t = 4$), but the prostate cancer subjects have a much higher risk of dying from other causes at that point. This observation is, at least in part, explained by differences in the respective age distributions; for instance, 93% of the prostate cancer subjects considered were over 50 years of age when diagnosed, while only 44% of the patients were older than 50 years at the time of diagnosis in the melanoma cohort.

**Fig. 2.**
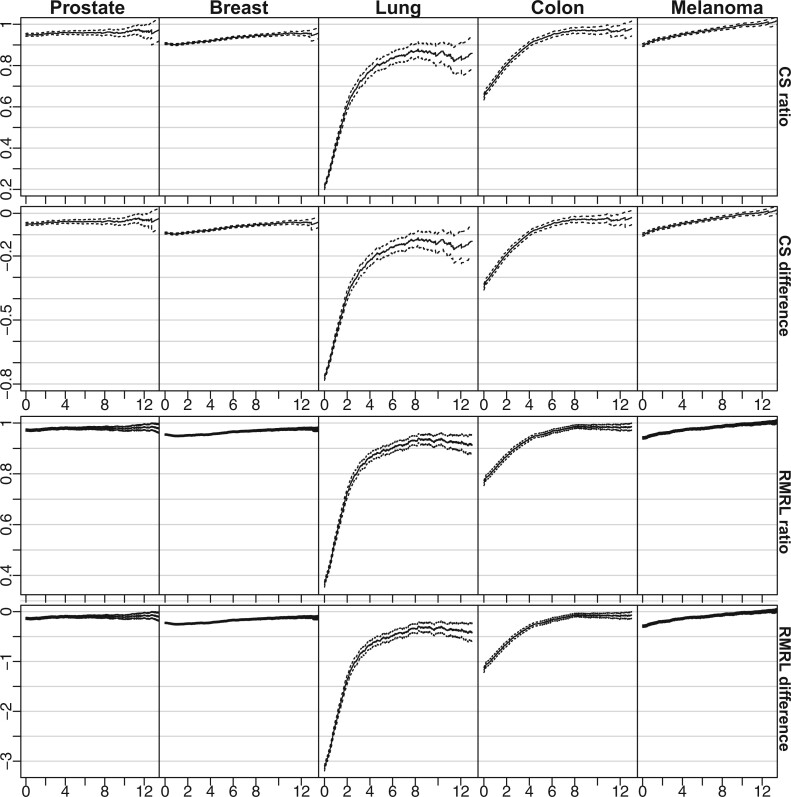
Estimates of the parameters (8)–(11) (ordered row-wise) with $\Delta = 5$ using the estimators described in Section 3. The dashed lines indicate 95% confidence intervals. The five cancers are ordered column-wise. The ticks at the $x$-axes are vertically aligned. The alert reader may have noticed that the upper two curves’ shapes and the lower two curves are remarkably similar within each cancer. This similarity is due to the roughly constant survival in the general population throughout the study period, obtained by matching wrt subjects aged $\leq 60$ when diagnosed. A roughly constant population survival yields that (8) and (9), as well as (10) and (11), are approximately proportional.

The parameters from Section 2.2 are plotted in Figure 2. We see that individuals with prostate cancer have a conditional 5-year survival ratio of about $0.95$ throughout the follow-up period, indicating that the prognosis of the prostate cancer subjects is good, but not improving over time. The prognosis of all the other cancer survivors improves over time, with breast, lung, and colon reaching a stable level 8–10 years after diagnosis. Plots of the conditional 5-year survival ratios and RMRL differences for the selected cancer cohorts are shown in Figure 2.

There is considerable improvement in the prognosis of lung and colon cancer survivors over time; the conditional 5-year survival ratios increase from 0.2 (95% CI within [0.19–0.21]) and 0.65 (95% CI [0.63–0.67]) at $t=0$ to around 0.87 and 0.97 (95% CI [0.84–0.91] and [0.95–0.98]), respectively, after 8 years of follow-up. After around 13 years, melanoma subjects have an estimated conditional 5-year survival ratio larger than one (95% CI [1.00–1.03]). We cannot give a causal interpretation of these trends. However, one reason may be differences in socioeconomic status: melanoma subjects have disproportionately high socioeconomic status, which in turn is associated with long survival (Idorn and Wulf, 2014). Similarly, subjects with lung cancer have disproportionately low socioeconomic status (Hovanec *and others,* 2018), which may be a reason why the parameters for the lung cancer cohort stop improving after around 6 years. Another reason may be a frailty phenomenon (Aalen, 1994; Stensrud *and others,* 2017).

If we assume that the true curves in Figure 2 are monotonically improving, there is a unique point when a given level of prognosis is achieved. In Table 1, we display the time until level of prognosis is reached, as defined by the conditional 5-year survival ratio (8).

**Table 1.** Estimates of the time until the desired prognosis $\delta$ is achieved, where $\delta$ is a fraction of the conditional 5-year survival ratio (the 95th percentiles are shown in parentheses)

Cancer	$\delta = 0.6$	$\delta = 0.7$	$\delta = 0.8$	$\delta = 0.9$	$\delta = 0.95$	$\delta = 1$
Prostate					1 (2.3)	—
Breast					9.7 (10.9)	—
Lung	2 (2.1)	2.9 (3.1)	4.5 (5.7)	—	—	—
Colon		0.6 (0.7)	2 (2.1)	3.9 (4.1)	6 (6.9)	—
Melanoma				0.1 (0.4)	3.9 (4.6)	11.2 (12.4)

The estimates are obtained by reading off the first time the estimates (percentiles) reach the horizontal lines in the second column of Figure 2. The empty entries indicate that the prognosis is achieved at $t=0$, while “$-$” indicates that the prognosis is not achieved during the follow-up period. From the table, we see that, for instance, the length of time until the 5-year conditional survival of the lung cancer cohort reached 60% of the 5-year conditional survival of the general population is 2 years, with the 95th percentile at 2.1 years. Prostate and breast cancer patients have a 5-year survival within 90% of the 5-year survival of the general population at the time of diagnosis. However, they differ in that it takes 1 (2.3) years for the prostate cancer subjects to reach 95% of the 5-year survival of the general population. In contrast, it takes as long as 9.7 (10.9) years for breast cancer subjects to reach 95% of the general population’s 5-year survival. The melanoma subjects got a 5-year survival equal to that of the general population after 11.2 (12.4) years.

## 5. Discussion

We have defined and described time-varying prognosis parameters that are relevant to doctors and patients. Furthermore, we have developed a general analytical variance estimator, which we explicitly express for all the parameters in this article. Importantly, if researchers come up with other parameters that fit into our ODE framework, they can use our method for estimation. We highlight that the covariance estimator can then be used without extra effort.

Our prognosis parameters are either (i) obtained using contrasts between all-cause mortality summary measures of the cancer cohort and the general population or (ii) using the information on cause-specific mortality in cohorts of cancer patients. To calculate the parameters in the first category, we must measure demographic variables associated with death in the general population. In particular, to consider patient-centered predictions, it would be desirable to include detailed demographic and lifestyle variables in both the cancer cohort and the general population. The second category of parameters is obtained without relying on detailed mortality tables. However, several of them require information on the causes of death. Exceptions are the conditional survival function and the restricted mean residual lifetime function, which provide notions of prognosis that do not distinguish between causes of death. In practice, it may be difficult to obtain reliable and detailed mortality tables, and causes of death, but there are exceptions, for example, Norwegian quality registries.

The parameters from Section 2.2 are inspired by previous work on relative survival, which are often used in analyses of cancer registry data (Belot *and others,* 2019; Mariotto *and others,* 2014). Previous work has aimed to disentangle the impact of cancer death and other death on survival when the cause of death information is lacking or is unreliable (Belot *and others,* 2019; Pohar Perme *and others*, 2012, 2016). Assuming the observed hazard in the cancer cohort decomposes into a population hazard (which depends on the measured demographic variables), and an excess hazard, estimators based on population tables can be derived (Pohar Perme *and others*, 2012, 2016). However, this decomposition can be invalid in real life, for example, due to lifestyle changes after getting a diagnosis, early detection of other diseases during follow-up, or frailty effects in the surviving cancer population. We do not rely on this decomposition; the parameters in Section 2.2 merely compare predicted outcomes in the cancer cohort with outcomes in a subgroup of the general population with similar demographic characteristics.

We have not targeted patient-centered predictions; such predictions can for example, be obtained by stratifying the parameters (1)–(11) on prognostic variables of interest, such as age, or stage. However, we expect that our predictions are increasingly patient-centered over time, as the survivors get more and more similar concerning prognostic variables over time due to frailty. Thus, time survived is both a prognostic variable and a proxy for all the (possibly unobserved) factors associated with death. Heuristically, by conditioning on survival, we implicitly “model” all such prognostic variables without imposing any modeling assumptions. Stratified analyses based on demographic variables such as age may be more desirable when comparing the cancer survivors with the general population. This is because age could have a different effect on survival in the two groups over time (e.g., due to side effects of cancer/cancer treatment getting more pronounced with increased age), leading to a distorted comparison of the groups over time. The parameters (1)–(7) are not subject to such differences. In applications, we think it is desirable to use several of the parameters considered here at the same time and that they together can provide valuable insight into the prognosis of the cancer patients.

We emphasize that our parameters are meant to give predictive information, and they cannot be interpreted causally, even in ideal randomized experiments. Indeed, the parameters are defined conditional on survival and thus they are prone to selection bias due to conditioning on a collider, analogously to hazard ratios (Aalen *and others,* 2015; Hernán, 2010). The parameters (1)–(11) should therefore not be understood as effects of cancer/cancer treatment on survival.

## Supplementary Material

kxab009_Supplementary_Data

## Software

Software used in Section 4.1 applied to similar simulated data sets can be found in the GitHub repository https://github.com/palryalen/paper-code.

## References

[B1] Aalen, O. (1994). Effects of frailty in survival analysis. Statistical Methods in Medical Research 3, 227–243. 7820293 10.1177/096228029400300303

[B2] Aalen, O. , Cook, R. and Røysland, K. (2015). Does cox analysis of a randomized survival study yield a causal treatment effect? Lifetime data analysis 21, 579–593. 26100005 10.1007/s10985-015-9335-y

[B3] Andersen, P. , Borgan, Ø., Gill, R. and Keiding, N. (1993). Statistical Models Based on Counting Processes. Springer Series in Statistics. New York: Springer.

[B4] Belot, A. , Ndiaye, A., Luque-Fernandez, M. A., Kipourou, D. K., Maringe, C., Rubio, F. J. and Rachet, B. (2019). Summarizing and communicating on survival data according to the audience: a tutorial on different measures illustrated with population-based cancer registry data. Clinical Epidemiology 11, 53–65. 30655705 10.2147/CLEP.S173523 PMC6322561

[B5] Ederer, F. , Axtell, L. and Cutler, S. (1961). The relative survival rate: a statistical methodology. Journal of National Cancer Institute Monographs 6, 101–121. 13889176

[B6] Hernán, M. (2010). The hazards of hazard ratios. Epidemiology 21, 13–15. 20010207 10.1097/EDE.0b013e3181c1ea43 PMC3653612

[B7] Hovanec, J. , Siemiatycki, J., Conway, D. I., Olsson, A., Stcker, I., Guida, F., Jckel, K., Pohlabeln, H., Ahrens, W., Brske, I., *and others*. (2018). Lung cancer and socioeconomic status in a pooled analysis of case-control studies. PLoS One 13, 1–18. 10.1371/journal.pone.0192999 PMC5819792 29462211

[B8] Idorn, L. W. and Wulf, H. C. (2014). Socioeconomic status and cutaneous malignant melanoma in Northern Europe. British Journal of Dermatology 170, 787–793. 24359255 10.1111/bjd.12800

[B9] Janssen-Heijnen, M. , Gondos, A., Bray, F., Hakulinen, T., Brewster, D. H., Brenner, H. and Coebergh, J. (2010). Clinical relevance of conditional survival of cancer patients in europe: age-specific analyses of 13 cancers. Journal of Clinical Oncology 28, 2520–2528. 20406936 10.1200/JCO.2009.25.9697

[B10] Janssen-Heijnen, M. , Houterman, S., Lemmens, V., Brenner, H., Steyerberg, E. and Coebergh, J. (2007). Prognosis for long-term survivors of cancer. Annals of Oncology 18, 1408–1413. 17693654 10.1093/annonc/mdm127

[B11] Larsen, I. , Møller, K., Johannesen, B., Robsahm, T. B., Grimsrud, T. E., Larønningen, T. K., Jakobsen, S. E. and Ursin, G. (2019). Cancer in Norway 2018 - Cancer incidence, mortality, survival and prevalence in Norway. Oslo: Cancer Registry of Norway.

[B12] Mariotto, A. B. , Noone, A. M., Howlader, N., Cho, H., Keel, G. E., Garshell, J., Woloshin, S. and Schwartz, L. M. (2014). Cancer survival: an overview of measures, uses, and interpretation. Journal of National Cancer Institute Monographs 2014, 145–186. 25417231 10.1093/jncimonographs/lgu024 PMC4829054

[B13] Merrill, R. M. (2018). Conditional relative survival among female breast cancer patients in the United States. Breast Journal 24, 435–437. 29063648 10.1111/tbj.12933

[B14] Pohar Perme, M. , Esteve, J. and Rachet, B. (2016). Analysing population-based cancer survival - settling the controversies. BMC Cancer 16, 933. 27912732 10.1186/s12885-016-2967-9 PMC5135814

[B15] Pohar Perme, M. and Pavlic, K. (2018). Nonparametric relative survival analysis with the r package relsurv. Journal of Statistical Software, Articles 87, 1–27.

[B16] Pohar Perme, M. , Stare, J. and Esve, J. (2012). On estimation in relative survival. Biometrics 68, 113–120. 21689081 10.1111/j.1541-0420.2011.01640.x

[B17] Ryalen, P. C. , Stensrud, M. J. and Rysland, K. (2018). Transforming cumulative hazard estimates. Biometrika 105, 905–916.

[B18] Stensrud, M. J. , Røysland, K. and Ryalen, P. C. (2019). On null hypotheses in survival analysis. Biometrics 75, 1276–1287. 31225636 10.1111/biom.13102

[B19] Stensrud, M. J. , Valberg, M., Røysland, K. and Aalen, O. O. (2017). Exploring selection bias by causal frailty models: the magnitude matters. Epidemiology 28, 379–386. 28244888 10.1097/EDE.0000000000000621

[B20] Wancata, L. M. , Banerjee, M., Muenz, D. G., Haymart, M. R. and Wong, S. L. (2016). Conditional survival in advanced colorectal cancer and surgery. Journal of Surgical Research 201, 196 – 201. 26850202 10.1016/j.jss.2015.10.021 PMC4744618

